# Low degree metabolites explain essential reactions and enhance modularity in biological networks

**DOI:** 10.1186/1471-2105-7-118

**Published:** 2006-03-08

**Authors:** Areejit Samal, Shalini Singh, Varun Giri, Sandeep Krishna, Nandula Raghuram, Sanjay Jain

**Affiliations:** 1 Department of Physics and Astrophysics, University of Delhi, Delhi 110007, India; 2 National Centre for Biological Sciences, UAS-GKVK Campus, Bangalore 560065, India; 3 School of Biotechnology, GGS Indraprastha University, Delhi 110006, India; 4 Jawaharlal Nehru Centre for Advanced Scientific Research, Bangalore 560064, India; 5 Santa Fe Institute, 1399 Hyde Park Road, Santa Fe, NM 87501, USA; 6 Niels Bohr Institute for Astronomy, Physics and Geophysics, Blegdamsvej 17, Copenhagen DK-2100, Denmark

## Abstract

**Background:**

Recently there has been a lot of interest in identifying modules at the level of genetic and metabolic networks of organisms, as well as in identifying single genes and reactions that are essential for the organism. A goal of computational and systems biology is to go beyond identification towards an explanation of specific modules and essential genes and reactions in terms of specific structural or evolutionary constraints.

**Results:**

In the metabolic networks of *Escherichia coli, Saccharomyces cerevisiae *and *Staphylococcus aureus*, we identified metabolites with a low degree of connectivity, particularly those that are produced and/or consumed in just a single reaction. Using flux balance analysis (FBA) we also determined reactions essential for growth in these metabolic networks. We find that most reactions identified as essential in these networks turn out to be those involving the production or consumption of low degree metabolites. Applying graph theoretic methods to these metabolic networks, we identified connected clusters of these low degree metabolites. The genes involved in several operons in *E. coli *are correctly predicted as those of enzymes catalyzing the reactions of these clusters. Furthermore, we find that larger sized clusters are over-represented in the real network and are analogous to a 'network motif. Using FBA for the above mentioned three organisms we independently identified clusters of reactions whose fluxes are perfectly correlated. We find that the composition of the latter 'functional clusters' is also largely explained in terms of clusters of low degree metabolites in each of these organisms.

**Conclusion:**

Our findings mean that most metabolic reactions that are essential can be tagged by one or more low degree metabolites. Those reactions are essential because they are the only ways of producing or consuming their respective tagged metabolites. Furthermore, reactions whose fluxes are strongly correlated can be thought of as 'glued together' by these low degree metabolites. The methods developed here could be used in predicting essential reactions and metabolic modules in other organisms from the list of metabolic reactions.

## Background

Evolution has produced organisms that are robust to various perturbations, yet the specific knockout of a single gene can be lethal to the organism. Similarly, organisms have some redundancy in their metabolic pathways, but single reactions whose knockout brings the growth of a cell to a halt – called 'essential' reactions – are also known to exist in metabolic networks [1-3]. What properties of a specific gene or reaction, within the context of the overall structure and organization of biochemical networks, make it essential for the organism? We show that most essential metabolic reactions in *Escherichia coli *[4], *Saccharomyces cerevisiae *[2] and *Staphylococcus aureus *[5] can be explained by the fact that they are associated with a low degree metabolite. Metabolic and protein interaction networks contain nodes with a large variation in their degree of connectivity [6-8]. In case of protein interaction networks it has been suggested that essentiality of a protein is correlated with its degree [8]. Hence, protein interaction networks are vulnerable to removal of highly connected proteins called 'hubs'. In contrast, for metabolic networks, one is usually interested in the essentiality of reactions rather than metabolites. Recently, Mahadevan and Palsson [9] have shown that low degree metabolites are almost as likely to be associated with essential reactions as high degree metabolites. We show here that in fact *almost all *essential reactions are explained by virtue of being tagged to some *low *degree metabolite.

Another theme in systems and computational biology has been to identify genetic regulatory modules [10-12], functional clusters [13-18] and graph-theoretic modules [19,20] in metabolic networks. Modularity of complex biological networks contributes to the robustness, flexibility, and evolvability of organisms, and also towards making their organization more comprehensible [21]. What structural features of metabolic networks cause specific subsets of metabolic reactions to have strongly correlated fluxes? We observe that low degree metabolites lead to one such structure in the metabolic network. Such metabolites contribute to a rigidity or coherence of reaction fluxes in the network resulting in clusters of highly correlated reactions. For example, in any steady state, where the concentrations of all metabolites are constant, a metabolite that can be produced in only one reaction and consumed in only one causes both reactions to have equal (or proportional with a fixed proportionality constant) fluxes. Maintaining the metabolic network close to a steady state then requires enzymes for both reactions to be simultaneously active, and hence the corresponding genes to be co-expressed, resulting in a transcription module containing those genes. In this work we first locate metabolites based purely on their low degree in the metabolic network. Then we show that clusters of their reactions predict genetic regulatory modules, as captured in the structure of operons [22,23], with a high probability in *E. coli*. Furthermore, the composition of most functional clusters is also explained via the low degree clusters embedded inside them. 

Biological networks have two properties that are currently regarded as unrelated: One, they have functional modules, and two, they have single genes or metabolic reactions whose knockout is lethal. An implication of the present work is that in metabolic networks, both properties can arise as consequences of the same structural property: the existence of low degree metabolites. Our work provides an *explanation*, rather than just identification, of essential reactions and metabolic modules.

### Lowest degree metabolites and their clusters

**Figure 1:**
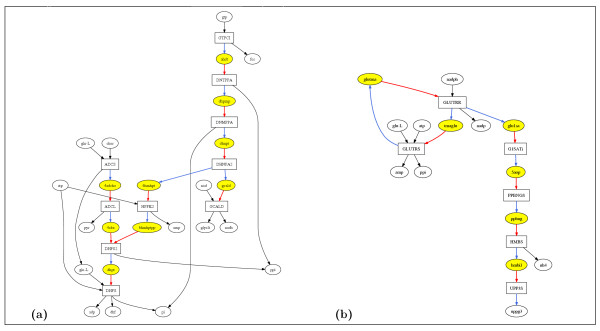
(a) UP-UC metabolites in the *E. coli *metabolic network forming a UP-UC cluster of 10 reactions. (b) UP-UC metabolites in the *S. aureus *metabolic network forming a UP-UC cluster of 6 reactions. Rectangles represent reactions and ovals metabolites. Yellow ovals represent UP-UC metabolites. Arrows to (from) metabolites represent their production (consumption) in reactions. A blue (red) link represents the production (consumption) of a UP (UC) metabolite. Note that UP-UC clusters are not strictly linear pathways. For example, in part (a) the reactions in the cluster are not all in a single chain and in part (b) there is a cycle inside the UP-UC cluster. Nevertheless fixing the flux of any one reaction in a UP-UC cluster fixes the fluxes of all other reactions in the cluster in any steady state, since the production rate of every UP-UC metabolite must be the same as its consumption rate. Hence, in part (a), fixing the flux of reaction GCALD fixes the flux of reaction DHNPA2 (because of the intermediate UP-UC metabolite gcald), which in turn fixes the fluxes of reactions HPPK2 and DNMPPA, and so on. All reactions in part (a) and (b) are globally essential in *E. coli *and *S. aureus *respectively. To reduce clutter, nodes corresponding to *h *(proton) and *h*_2_*o *have been omitted. Abbreviation of metabolite and reaction names in part (a) are as in [4] and in part (b) as in [5]. The figures have been drawn using Graphviz software.

A metabolite may be designated as 'uniquely produced' or 'UP' ('uniquely consumed' or 'UC') if, in the bipartite graph of reactions and metabolites, the node corresponding to the metabolite has in-degree (out-degree) equal to unity; in other words, if there is only one reaction in the network that produces (consumes) the metabolite. A metabolite that is both UP and UC (a 'UP-UC metabolite') has the lowest degree in the network. Examples of UP-UC metabolites taken from the metabolic networks [4,5] of *E. coli *and *S. aureus *can be seen in Fig. 1. In any metabolic steady state the concentration of a metabolite is fixed; its rate of production is equal to that of consumption. Hence for a UP-UC metabolite in any steady state, the flux of the reaction producing it is proportional to that of the reaction consuming it, with the proportionality constant determined by the stoichiometric coefficients of the metabolite in the two reactions. A 'UP-UC cluster' of reactions may be defined as a set of reactions connected by UP-UC metabolites. In a steady state fixing the flux of any reaction in the UP-UC cluster fixes the fluxes of all other reactions in the cluster (see Fig. 1). These clusters include linear pathways but can also have branched or cyclic structure. UP-UC clusters are special cases of reaction/enzyme subsets [13-15] and fully coupled reactions or co-sets [16-18]. UP-UC metabolites give rise to a situation wherein the inclusion of one reaction in a set implies the inclusion of another; such situations have also been considered [24-26] in the context of identification and generation of feasible pathways and applied to *E. coli *metabolism. Each UP-UC cluster of reactions can be replaced by an effective reaction without affecting the steady state performance and can be used to coarse-grain metabolic networks [13,14].

A reaction was designated as 'uniquely producing' or 'UP' ('uniquely consuming' or 'UC') if it produced (consumed) a UP (UC) metabolite. The number of UP (UC) reactions in the metabolic networks of *E. coli, S. cerevisiae *and *S. aureus *were found to be 289 (272), 391 (370) and 277 (218), respectively, while the number of reactions that are either UP or UC or both (we refer to this set as 'UP/UC reactions') is 417, 583 and 376. We will show below that such reactions play a special role in metabolic networks.

## Results

### Essential reactions are largely explained by UP/UC structure

We used the flux balance analysis (FBA) [1,27-29] approach to determine essential reactions in the metabolic networks of *E. coli, S. cerevisiae *and *S. aureus*. We computed the steady state optimal flux vectors for each of these organisms in aerobic conditions for all permissible single organic carbon sources in a minimal medium. We found a feasible solution (with a nonzero growth rate) for 89, 43 and 27 sources in *E. coli, S. cerevisiae *and *S. aureus *respectively. The list of feasible carbon sources under minimal media in these organisms is provided in Supplementary Tables S1, S2 and S3 (see Additional File 1). 

We considered the effect of 'switching off' reactions (by setting their maximum flux equal to zero) one by one, on the optimal growth rate for each food source. A reaction was designated as 'essential' for a particular food source if switching it off resulted in a zero optimal growth rate under that input condition. We designated a reaction as 'globally essential' for an organism if it was essential for all its feasible minimal media under aerobic conditions. The number of essential reactions for each minimal media varied between 200 and 240 reactions and the number of globally essential reactions was 164 for the *E. coli *metabolic network. Similarly, we found that the number of globally essential reactions in metabolic networks of *S. cerevisiae *and *S. aureus *were 127 and 196 respectively.

#### Most essential reactions either produce or consume a UP or UC metabolite

Of the 164 globally essential reactions in the *E. coli *metabolic network, 133 were found to be either UP or UC. The probability of such a high overlap occurring by pure chance is very small. We can quantify this by comparing to a null model in which the essentiality and the UP/UC property of a reaction are considered to be independent of each other. The probability that out of a set of 1176 reactions two independently chosen subsets of size 417 (= number of UP/UC reactions) and 164 (= number of globally essential reactions) will have an intersection of 133 or greater is *p <*10^-37 ^(any one or both of the subsets is chosen randomly). Similarly, we found a high fraction of globally essential reactions in metabolic networks of *S. cerevisiae *and *S. aureus *to be UP or UC (see Table 1). This explains why this subset is essential: there is simply no other path around these reactions in the entire network to produce or consume some metabolite that is presumably required for the eventual production of biomass. In a recent paper [9] Mahadevan and Palsson have determined, for each metabolite in the network, the fraction of its reactions that are essential. They have observed that this 'lethality fraction' of the low degree metabolites is on average comparable to high degree metabolites, and in particular, some metabolites with in and out degree unity (that we have designated here as UP-UC metabolites) have lethality fraction unity. We present here a stronger result regarding the role of low degree metabolites: most essential reactions involve at least one UP or UC metabolite. These reactions may involve other metabolites of higher degree, but their essentiality is due to their uniqueness in producing or consuming a low degree metabolite.

#### The correspondence between essential and UP/UC reactions is even tighter in the 'reduced network'

To understand the remaining globally essential reactions, we considered a reduced or pruned version of the network. Certain reactions in various reconstructed metabolic networks are such that they have a zero flux value under all steady states for stoichiometric reasons. These reactions are referred to as 'strictly detailed balanced' reactions [30] or 'blocked' reactions [17], and can be removed from the network for any steady state analysis. We used a previously described algorithm [17] to determine blocked reactions in the metabolic networks of *E. coli, S. cerevisiae *and *S. aureus*. We found 290 (800, 294) of the 1176 (1579, 865) reactions in the *E. coli *(*S. cerevisiae, S. aureus*) metabolic network to be blocked. We removed the blocked reactions from each network to obtain the 'reduced network' for each organism (containing 886, 779 and 571 reactions respectively).

Note that the essential reactions obtained by implementing FBA on the reduced network are exactly the same as those obtained from the original network for each input condition. Hence, instead of requiring a metabolite to be UP or UC across the entire metabolic network, we asked if it was UP or UC in the reduced network. The set of UP(UC) metabolites and reactions so obtained turns out to be somewhat smaller than the original set. In *E. coli, S. cerevisiae *and *S. aureus *the new set of UP/UC reactions has 352, 306 and 276 reactions. This is so because several reactions that were UP/UC in the original network happen to be blocked and are now removed. Conversely some metabolite that was earlier not UP(UC) can now become UP(UC) after the removal of a reaction. This adds new reactions to the UP/UC set but this number turns out to be smaller than the number removed (details are given in Supplementary Table S4 in Additional File 1). The new UP(UC) metabolites have, by definition, their in (out) degree unity in the reduced network; even in the original network they have a low degree (for *E. coli *their average in (out) degree in the original network is 1.31 (1.33)). We emphasize that the reduced network as defined above and hence the set of new UP(UC) reactions is uniquely determined by the original network.

We found that 156 out of the 164 globally essential reactions (95 %) in the *E. coli *metabolic network to be UP or UC in the reduced network (*p <*10^-62^). Similarly, we found that almost all globally essential reactions in *S. cerevisiae *and *S. aureus *were either UP or UC in the reduced network (92 and 93 % respectively; see Table 1) thereby underscoring the fact that nodes with a low degree of connectivity play an 'essential' role in metabolism. The importance of low-degree nodes in the essential functionality of complex autocatalytic networks has also been observed elsewhere [31] in a different context.

This finding provides some insight into the structural or topological origin of essential reactions in metabolic networks. It is, of course, obvious that if a certain metabolite is an essential intermediate for the production of some biomass metabolite, and if this metabolite is uniquely produced or uniquely consumed, then the corresponding production or consumption reaction will be essential for the growth of the cell. However the converse of this statement – that all essential reactions in the network should have this topological property – is far from obvious. Our finding that about 5–8 % of essential reactions do not have this property proves that the converse statement is indeed false. Thus the fact that the overwhelming majority (92–95 %) of essential reactions have this topological property is a characterization of the nature of metabolic networks found in organisms. We remark that we do not as yet understand why the remaining essential reactions happen to be essential.

#### Most UP/UC reactions are essential in some condition or other

We found that there are 352 UP or UC metabolic reactions in the *E. coli *reduced network. 156 of these 352 reactions were globally essential, while 288 of these 352 reactions (82 %) were essential for at least one of the 89 possible minimal media in *E. coli*. Such a large overlap is very unlikely (*p <*10 ^-74^), given that the number of reactions that are essential for at least one of the input conditions in the reduced *E. coli *metabolic network is 400. Some of these UP/UC reactions were part of the input pathways of only one carbon source, hence they were essential only for that input. In *S. cerevisiae *170 out of 306 UP/UC reactions (56 %) in the reduced network are essential in at least one input condition, while in *S. aureus *257 out of 276 (93 %) have this property. The *p *values for such large overlaps in the two organisms are, respectively, *p <*10^-22 ^and *p <*10^-67^, given that the number of reactions that are essential for at least one of the input conditions in those networks is 269 and 331. The substantial difference between *S. cerevisiae*, a eukaryote, and the two bacteria may reflect a more evolved metabolic structure that needs to be further investigated.

#### Comparison between computationally determined essential reactions and lethal single gene knockouts

To check the agreement of essential reactions in the *E. coli *metabolic network with a database [32] of experimentally determined essential genes in a rich medium, we implemented FBA for a rich medium containing all food sources for the *E. coli *metabolic network [33]. We found 95 reactions to be essential in this medium for *E. coli*. 89 of these 95 reactions were found to be either UP or UC in the reduced network. Of the 95 essential reactions in rich medium, information about the corresponding genes was available for only 85 reactions. Of these 85, 14 reactions had known isozymes, i.e, multiple enzymes catalyzing the reaction, hence the corresponding genes are not expected to be essential. Of the remaining 71 reactions, 5 had associated genes whose essentiality was undetermined in the database. Of the remaining 66 reactions, 38 reactions had associated genes that had been found to be essential in the database [32], which is a fairly high fraction. Conversely, of the 618 essential genes determined for *E. coli *by Gerdes et al, 158 genes were also part of the *E. coli *metabolic network [4] used for our study. 103 of the above 158 essential genes had their products catalyzing only a single reaction in the *E. coli *metabolic network. Of these 103 essential genes, 62 were associated with a UP or UC reaction. Further, using the reduced network, we found that 73 of the 103 essential genes were associated with a UP or UC reaction. The discrepancy between theoretical prediction and experimental data may be reconciled by the incomplete knowledge about possible isozymes for certain reactions or uncharacterized alternative metabolic pathways in the present in-silico metabolic model [3].

### Low degree clusters predict regulatory modules

**Figure 2:**
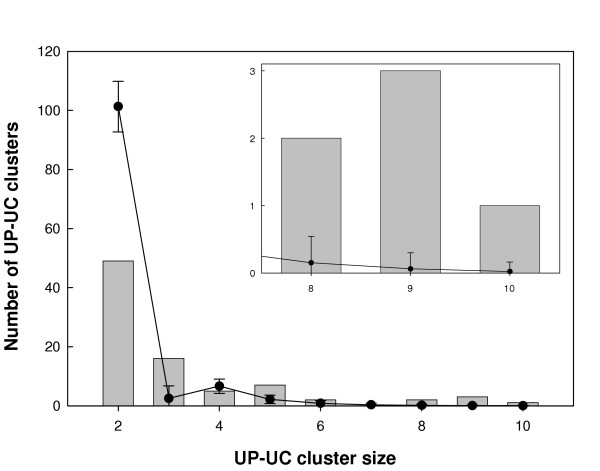
Frequency histogram of UP-UC cluster sizes in the *E. coli *metabolic network (grey bars). Data is shown in Supplementary Table S5 (see Additional File 1). The black line is the frequency distribution for the randomized versions of the network (averaged over 1000 realizations) that preserve the in- and out-degree of all nodes. Error bars show one standard deviation of the randomized ensemble. **Inset: **Enlargement of the graph for the larger sized clusters. In the real network, larger UP-UC clusters (size ≥ 8) occur much more often than in the randomized version (*p *< 0.001). On the other hand, smaller UP-UC clusters (size ≤ 3) occur much less often than in the randomized version (*p *< 0.001).

We found that the *E. coli *metabolic network [4] contained 185 UP-UC metabolites. We determined all UP-UC clusters in the network (see methods). The total number of UP-UC clusters in *E. coli *metabolic network was found to be 85; their size distribution is shown by the grey bars in Fig. 2. The list of all reactions in each UP-UC cluster for the *E. coli *metabolic network is given in Supplementary Table S6 (see Additional File 1). We then investigated whether the genes coding for the enzymes of the reactions in a UP-UC cluster are part of the same operon in *E. coli*. Genes on the same operon are by definition part of a genetic module since they are coregulated. At the moment genes corresponding to enzymes of reactions of the network have been identified for only part of the network. Of the 85 UP-UC clusters in the *E. coli *metabolic network, only 69 clusters had two or more reactions with known corresponding genes. We looked at the regulation of these 69 UP-UC clusters using the known operon information from RegulonDB [22] and Ecocyc [23] databases. Genes (of reactions within UP-UC clusters) that belong to the same operon in *E. coli *are indicated in Supplementary Table S6 (see Additional File 1). For 42 of the 69 UP-UC clusters, we found that two or more genes of the cluster were part of the same operon. Further, 36 of these 42 UP-UC clusters had at least half of their genes belonging to the same operon. We also found that 21 UP-UC clusters have at least one possible set of constituent genes catalyzing all reactions in the cluster belonging to the same operon.

To show that two genes belonging to a UP-UC cluster in *E. coli *have greater probability of lying on the same operon than otherwise expected, we performed the following test. We found 251 unique genes catalyzing various reactions in the 69 UP-UC clusters. If we randomly pick any two of these 251 genes, the probability that the two genes lie on the same operon is 0.0057. If we randomly pick a pair of genes that belong to the same UP-UC cluster from this set of 251 genes, the probability that the two genes lie on the same operon is 0.29. Thus regulatory modules are predicted correctly with a high probability by this method. It is possible that UP-UC clusters will find even greater correspondence with regulatory modules when expression data is analysed; our comparison rests only on operon data, and only about 25 percent of the transcriptional regulatory network of *E. coli *is presently believed to have been identified [3]. It would also be interesting to extend this analysis to the other two organisms.

### Large UP-UC clusters are analogous to network motifs

We asked the question: Is it expected that a network like the *E. coli *metabolic network of 618 metabolites and 1176 reactions with 185 UP-UC metabolites will have a distribution of UP-UC clusters as given in Figure 2? To answer this question, we compared the distribution of UP-UC clusters in the real *E. coli *metabolic network with a suitably randomized version of the network [34]. The randomized network has the same number of metabolite nodes and reaction nodes and the same number of incoming and outgoing links at each node as the real *E. coli *metabolic network (see methods). Averaging over 1000 realizations of the randomized metabolic network we found a cluster distribution as shown by the black line in Fig. 2. This shows that the actual metabolic network of *E. coli *has its UP-UC metabolites bunched up next to each other, forming larger clusters than expected in random networks with the same local connectivity properties. Thus, larger size (size ≥ 8) UP-UC clusters are over-represented in the real *E. coli *metabolic network, and may be collectively considered as analogous to a network *motif *(as defined in [34,35]), while smaller size (≤ 3) UP-UC clusters are under-represented in the real network, and may be collectively considered analogous to an 'anti-motif' [36]. We also found qualitatively similar results for the metabolic networks of *S. cerevisiae *and *S. aureus *(data not shown).

### Low degree metabolites explain perfect clusters

Correlated reaction sets are sets of reactions in the metabolic network that are always used together in functional states of the network. Each flux vector obtained using FBA represents one possible functional state of the network. For each feasible minimal medium we obtained one flux vector with a nonzero growth rate. We defined an 'active' reaction as one that had a nonzero flux in at least one of the latter flux vectors. Then we computed the correlation coefficient among fluxes of the active reactions across these flux vectors in a manner analogous to the correlation of gene activity from microarray data across different conditions [10] (see methods). A 'perfect cluster' is a set of reactions whose pairwise correlation coefficients with each other are all unity across all sets of conditions. Reactions in perfect clusters have fluxes that are proportional to each other with the same proportionality constant under all the flux vectors considered.

We found that in the *E. coli *metabolic network, most of the 582 active reactions under 89 input conditions were contained in several perfect clusters of size 2 or more (see Table 2). These clusters, reported earlier in [37] overlap highly with the clusters of [18]. One might ask: Why are particular subsets of reactions perfectly clustered to each other. UP-UC clusters provide a structural explanation for these perfect clusters. Of the 85 UP-UC clusters in the entire *E. coli *network, 46 UP-UC clusters are in the set of active reactions. All the 46 active UP-UC clusters are subsets of perfect clusters. To further explain the observed clustering of reactions in the *E. coli *metabolic network, we considered UP(UC) metabolites in the reduced network. We found 94 UP-UC clusters in the reduced network for *E. coli*. Table 2 shows that most of the perfect clusters in *E. coli *are explained in terms of UP-UC clusters in the reduced network in the sense that UP-UC clusters account for the bulk of reactions in the perfect clusters. Most of the co-sets reported in [18] for *E. coli *are also explained by UP-UC clusters in the reduced network (see Supplementary Table S7 in Additional File 1). Further, we found that most perfect clusters in the metabolic networks of *S. cerevisiae *and *S. aureus *are also explained by UP-UC clusters in their respective reduced networks (see Supplementary Tables S8 and S9 in Additional File 1).

Very recently Almaas *et al *[38] have observed a set of 90 reactions that are always active under a diverse set of 30000 conditions in the *E. coli *metabolic network that they designate as the 'core' of the network. They also find these reactions to be highly correlated and most of them (81 in number) to be essential. We find that 79 of these 90 reactions are UP/UC (*p <*10^-19^).

## Discussion and Conclusion

In this paper we have observed that the lowest degree metabolites are implicated in two distinct properties of the metabolic networks, one, the existence of essential metabolic reactions (and lethal single metabolic gene knockouts), and two, existence of functional clusters in the metabolic networks (and associated regulatory modules).

To some extent the identification of UP/UC metabolites depends on the way the metabolic network is curated. For example, the networks we have used leave out certain non-enzymatic reactions such as protonation-deprotonation reactions. Since their inclusion would render some of the presently UP(UC) metabolites non-UP(UC), our definition of UP(UC) could be criticized as being somewhat arbitrary. In this context it is worth noting that for the networks as they stand, our definition of UP(UC) allows us to establish a connection between distinct properties of the network (e.g., between essentiality, a functional property and the UP/UC character, a topological property), and that our main findings hold for metabolic networks of three distinct organisms. This suggests that UP/UC reactions as defined by us do capture a certain pattern. In our view the important point is not that other definitions of the network would obscure the pattern, but rather, that there do exist systematic definitions of the network in which a pattern is visible.

In metabolic networks the very existence of essential reactions is an indicator of the fragility of the system: Even though the network has many reaction nodes, the removal of a single essential reaction node destroys the functionality of the network completely by blocking the flow of an essential intermediate. Isozymes are a way of dealing with this fragility. However, not all essential reactions have isozymes [39]; this means that evolution has tolerated this fragility. Our finding that essential reactions are tagged by low degree metabolites may provide some insight into why this is the case. Metabolites that participate in very few reactions perhaps do so in part because some feature of their chemical structure prohibits ready association with other molecules, i.e., their low degree is a consequence of constraints coming from chemistry. Then evolution tolerates the reactions that produce or consume such metabolites as essential because chemistry leaves it no choice.

Alternatively, it could be that this fragility happens to be a byproduct of some other desirable structural property that contributes to robustness or evolvability, such as modularity. We have drawn attention to the fact that low degree metabolites also play a role in functional clustering of reactions in the metabolic network. We have further provided evidence that the UP-UC clusters at the metabolic level correspond, with a high probability, to sets of genes forming modules at the regulatory level in *E. coli*.

This raises the question: if low degree metabolites contribute to modularity, could it be that the evolutionary advantages of that have outweighed the disadvantage of the above mentioned fragility caused by the same low degree metabolites? Is it the case that evolution has preferred 'chemically constrained' low degree metabolites in spite of the fragility they cause because they contribute to modularity? A goal in biology is to understand highly evolved biological organization in terms of simpler and more inevitable structures [40]. Here we have presented evidence that certain genetic regulatory modules, in particular certain operons, mirror the low degree structure of the metabolites whose production and consumption they regulate. This could be an example of how the origin of certain regulatory structure can be traced to simple chemical constraints.

## Methods

### Detection of UP-UC clusters

We used recently reconstructed metabolic networks of *E. coli *(version iJR904 [4]), *S. cerevisiae *(version iND750 [2]) and *S. aureus *(version iSB619 [5]) in this study. The networks were downloaded from the website [41]. Each reversible reaction in the network was converted into two one sided reactions. We excluded the external metabolites in the three metabolic networks while determining the UP-UC metabolites. For calculating various UP-UC clusters, we first identify all UP-UC metabolites in the bipartite graph of the network. We then delete all links in the graph except those going into and out of UP-UC metabolites. From this new bipartite graph, we generate a reaction-reaction graph, in which two reactions are connected if one consumes a metabolite produced by the other. The weak components of size ≥ 2 of the reaction-reaction graph are the various UP-UC clusters in the network. An algorithm for computing UP-UC clusters is given in the Additional File 2.

### Generation of randomized networks

We constructed the matrix *A = *(*A*_*iα*_) where *A*_*iα *_equals 1 if metabolite *i *is produced in reaction *α*, -1 1if it is consumed in reaction *α *and 0 if it does not participate in reaction *α*. *A *is an *n *× *N *matrix, where *n *is the number of internal metabolites in the network and *N *is the number of reactions. In the above mentioned networks of the three organisms *E. coli, S. cerevisiae *and *S. aureus*, we have (*n,N*) = (618,1177), (945,1580), (561,866) respectively. This includes the biomass reaction. Each nonzero entry of *A *defines a link in the bipartite graph of metabolites and reactions. Starting from *A *for the real network, we generated randomized networks keeping the degree of each metabolite and reaction node unchanged [42,43]. It is important to distinguish between two kinds of links; one coming into a metabolite node from a reaction node and the other going out of a metabolite node to a reaction node. All the links or edges in this bipartite graph were divided into these two groups. Two links are then randomly selected in one of these two groups and swapped. Before swapping, we ensure that the metabolite involved in any link is not already involved in the reaction corresponding to the other link. Furthermore, links corresponding to the biomass reaction are not picked for swapping. This process of selecting a random pair of links was repeated 18000 times. We verified that more than 99.9% of the links were visited at least once. Starting from the real metabolic network, this procedure is repeated 1000 times (with different random number seeds), the UP-UC clusters determined for each of the 1000 realizations of the randomized network and the average taken thereof.

### Perfect clusters

Using FBA we obtained vαI
 MathType@MTEF@5@5@+=feaafiart1ev1aaatCvAUfKttLearuWrP9MDH5MBPbIqV92AaeXatLxBI9gBaebbnrfifHhDYfgasaacH8akY=wiFfYdH8Gipec8Eeeu0xXdbba9frFj0=OqFfea0dXdd9vqai=hGuQ8kuc9pgc9s8qqaq=dirpe0xb9q8qiLsFr0=vr0=vr0dc8meaabaqaciaacaGaaeqabaqabeGadaaakeaacqWG2bGDdaqhaaWcbaacciGae8xSdegabaGaemysaKeaaaaa@310F@, the velocity of reaction *α *in an optimal steady state corresponding to input condition *I*, *I *= 1,..., *M*, *α *= 1,..., *N*, where *M *is the number of feasible minimal media and *N *is the number of distinct one way reactions in the metabolic network. These vαI
 MathType@MTEF@5@5@+=feaafiart1ev1aaatCvAUfKttLearuWrP9MDH5MBPbIqV92AaeXatLxBI9gBaebbnrfifHhDYfgasaacH8akY=wiFfYdH8Gipec8Eeeu0xXdbba9frFj0=OqFfea0dXdd9vqai=hGuQ8kuc9pgc9s8qqaq=dirpe0xb9q8qiLsFr0=vr0=vr0dc8meaabaqaciaacaGaaeqabaqabeGadaaakeaacqWG2bGDdaqhaaWcbaacciGae8xSdegabaGaemysaKeaaaaa@310F@ define the *M *flux vectors we consider. A reaction *α *is said to be active if vαI
 MathType@MTEF@5@5@+=feaafiart1ev1aaatCvAUfKttLearuWrP9MDH5MBPbIqV92AaeXatLxBI9gBaebbnrfifHhDYfgasaacH8akY=wiFfYdH8Gipec8Eeeu0xXdbba9frFj0=OqFfea0dXdd9vqai=hGuQ8kuc9pgc9s8qqaq=dirpe0xb9q8qiLsFr0=vr0=vr0dc8meaabaqaciaacaGaaeqabaqabeGadaaakeaacqWG2bGDdaqhaaWcbaacciGae8xSdegabaGaemysaKeaaaaa@310F@ > 0 for some *I*. Given a set of *M *flux vectors, the correlation coefficient [10] between two active reactions *α *and *β *is given by

Cαβ=(1/M)∑I=1MvαIvβI/(φαφβ),
 MathType@MTEF@5@5@+=feaafiart1ev1aaatCvAUfKttLearuWrP9MDH5MBPbIqV92AaeXatLxBI9gBaebbnrfifHhDYfgasaacH8akY=wiFfYdH8Gipec8Eeeu0xXdbba9frFj0=OqFfea0dXdd9vqai=hGuQ8kuc9pgc9s8qqaq=dirpe0xb9q8qiLsFr0=vr0=vr0dc8meaabaqaciaacaGaaeqabaqabeGadaaakeaacqWGdbWqdaWgaaWcbaacciGae8xSdeMae8NSdigabeaakiabg2da9iabcIcaOiabigdaXiabc+caViabd2eanjabcMcaPmaaqahabaGaemODay3aa0baaSqaaiab=f7aHbqaaiabdMeajbaakiabdAha2naaDaaaleaacqWFYoGyaeaacqWGjbqsaaGccqGGVaWlcqGGOaakcqWFgpGzdaWgaaWcbaGae8xSdegabeaakiab=z8aMnaaBaaaleaacqWFYoGyaeqaaOGaeiykaKcaleaacqWGjbqscqGH9aqpcqaIXaqmaeaacqWGnbqta0GaeyyeIuoakiabcYcaSaaa@50AA@

where φα=[(1/M)∑I=1M(vαI)2]1/2
 MathType@MTEF@5@5@+=feaafiart1ev1aaatCvAUfKttLearuWrP9MDH5MBPbIqV92AaeXatLxBI9gBaebbnrfifHhDYfgasaacH8akY=wiFfYdH8Gipec8Eeeu0xXdbba9frFj0=OqFfea0dXdd9vqai=hGuQ8kuc9pgc9s8qqaq=dirpe0xb9q8qiLsFr0=vr0=vr0dc8meaabaqaciaacaGaaeqabaqabeGadaaakeaaiiGacqWFgpGzdaWgaaWcbaGae8xSdegabeaakiabg2da9iabcUfaBjabcIcaOiabigdaXiabc+caViabd2eanjabcMcaPmaaqadabaGaeiikaGIaemODay3aa0baaSqaaiab=f7aHbqaaiabdMeajbaakiabcMcaPmaaCaaaleqabaGaeGOmaidaaaqaaiabdMeajjabg2da9iabigdaXaqaaiabd2eanbqdcqGHris5aOGaeiyxa01aaWbaaSqabeaacqaIXaqmcqGGVaWlcqaIYaGmaaaaaa@48C9@. Reactions *α *and *β *are said to be perfectly correlated in the given set of flux vectors if *C_αβ _*= 1 for that set and all its subsets of flux vectors. A numerical value of *C_αβ _*≥ 0.999999 was taken as 'unity' for this purpose. Perfect clusters were identified by locating maximal sets of reactions that were perfectly correlated to each other pairwise.

## Supplementary Material

Additional File 1 Supplementary Tables S1 to S9. Click here for file

Additional File 2 Algorithm for computing UP-UC clusters in the metabolic network. Click here for file

## Figures and Tables

**Table 1:** Almost all globally essential reactions in *E. coli, S. cerevisiae *and *S. aureus *are UP or UC. The *p *value represents the probability that the indicated overlap would arise in a null model as discussed in the text.

**Organism**	*E. coli*	*S. cerevisiae*	*S. aureus*
Total number of reactions	1176	1579	865
Number of globally essential reactions	164	127	196
Number of globally essential reactions that are UP or UC in the entire network	133 (*p *< 10^-37^)	86 (*p *< 10^-12^)	157 (*p *< 10^-32^)
Number of globally essential reactions that are UP or UC in the reduced network	156 (*p *< 10^-62^)	117 (*p *< 10^-41^)	182 (*p *< 10-^58^)

**Table 2:** The size distribution of perfect clusters in the *E. coli *metabolic network and their explanation in terms of UP-UC clusters. The third column lists the number of perfect clusters that are explained by UP-UC clusters calculated using the reduced network. The fourth column gives the breakup of the explained perfect clusters in terms of UP-UC clusters of various sizes. E.g. in the second row the entry 7 × (3) + 2 × (2) implies that 7 UP-UC clusters of size 3 are identical to 7 perfect clusters of size 3 and furthermore, two UP-UC clusters of size 2 are subsets of two size 3 perfect clusters. In the fourth row the term 1 × (3+2) means that one of perfect clusters of size 5 contained two distinct UP-UC clusters of sizes 3 and 2. There are 26 UP-UC clusters that are part of the largest perfect cluster of 148 reactions accounting for 125 reactions in it. This largest perfect cluster is a subset of reactions that are active for all input conditions and is located near the output end of the metabolic network.

Size of perfect clusters	Number of perfect clusters	Number of perfect clusters explained	Breakup of explained clusters into UP-UC clusters in the reduced network
2	48	22	22 × (2)
3	19	9	7 × (3) + 2 × (2)
4	11	10	8 × (4) + 1 × (3) + 1 × (2)
5	4	3	1 × (4) + 1 × (3+2) + 1 × (2+2)
6	1	1	1 × (6)
7	1	1	1 × (7)
8	2	2	1 × (6+2) + 1 × (5+2)
148	1	1	(14+12+10+9+7+6+6+6+5+5+4+4+4+4+4+3+3+3+2+2+2+2+2+2+2+2)
