# Supplementary material for: Low degree metabolites explain essential reactions and enhance modularity in biological networks
Source: BMC Bioinformatics. 2006 Mar 8;7:118. doi: 10.1186/1471-2105-7-118 (PMC1434774; doi:10.1186/1471-2105-7-118)
Supplement: Additional File 1 — Supplementary Tables S1 to S9. [file 1471-2105-7-118-S1.pdf]

Additional File 1

(Supplementary Tables S1 to S9)

for

"Low degree metabolites explain essential reactions and enhance modularity in biological networks"

by

Areejit Samal, Shalini Singh, Varun Giri, Sandeep Krishna, Nandula Raghuram and Sanjay Jain

Supplementary Table S1: List of carbon sources that gave flux vectors with a nonzero growth rate under minimal media for aerobic conditions in the *E. coli* metabolic network (version iJR904; Reed *et*. *al*. *Genome Biology* **4**: R54.1 (2003))

Abbreviation	Metabolite name	Abbreviation	Metabolite name
12ppd-S	(S)-Propane-1,2-diol	glu-L	L-Glutamate
2ddglcn	2-Dehydro-3-deoxy-D-gluconate	gly	Glycine
3hcinnm	3-hydroxycinnamic acid	glyald	D-Glyceraldehyde
3hpppn	3-(3-hydroxy-phenyl)propionate	glyc	Glycerol
4abut	4-Aminobutanoate	glyc3p	Glycerol 3-phosphate
ac	Acetate	glyclt	Glycolate
acac	Acetoacetate	gsn	Guanosine
acald	Acetaldehyde	hdca	Hexadecanoate (n-C16:0)
acgam	N-Acetyl-D-glucosamine	idon-L	L-Idonate
acmana	N-Acetyl-D-mannosamine	ins	Inosine
acnam	N-Acetylneuraminate	lac-D	D-Lactate
adn	Adenosine	lac-L	L-Lactate
akg	2-Oxoglutarate	lcts	Lactose
ala-D	D-Alanine	mal-L	L-Malate
ala-L	L-Alanine	malt	Maltose
alltn	Allantoin	malthx	Maltohexaose
arab-L	L-Arabinose	maltpt	Maltopentaose
arg-L	L-Arginine	malttr	Maltotriose
asn-L	L-Asparagine	maltttr	Maltotetraose
asp-L	L-Aspartate	man	D-Mannose
but	Butyrate (n-C4:0)	man6p	D-Mannose 6-phosphate
cit	Citrate	melib	Melibiose
cytd	Cytidine	mnl	D-Mannitol
dad-2	Deoxyadenosine	ocdca	octadecanoate (n-C18:0)
dcyt	Deoxycytidine	orn	Ornithine
dgsn	Deoxyguanosine	pppn	Phenylpropanoate
dha	Dihydroxyacetone	pro-L	L-Proline
din	Deoxyinosine	ptrc	Putrescine
duri	Deoxyuridine	pyr	Pyruvate
etoh	Ethanol	rib-D	D-Ribose
fru	D-Fructose	rmn	L-Rhamnose
fuc-L	L-Fucose	sbt-D	D-Sorbitol
fum	Fumarate	ser-D	D-Serine
g6p	D-Glucose 6-phosphate	ser-L	L-Serine
gal	D-Galactose	succ	Succinate
galct-D	D-Galactarate	sucr	Sucrose
galctn-D	D-Galactonate	tartr-L	L-tartrate
galt	Galactitol	thr-L	L-Threonine
galur	D-Galacturonate	tre	Trehalose
gam	D-Glucosamine	trp-L	L-Tryptophan
glc-D	D-Glucose	ttdca	tetradecanoate (n-C14:0)
glcn	D-Gluconate	uri	Uridine
glcr	D-Glucarate	xtsn	Xanthosine
glcur	D-Glucuronate	xyl-D	D-Xylose
gln-L	L-Glutamine		

Each carbon source was provided along with ammonium, Fe2+, oxygen, phosphate, potassium, proton, sodium, sulfate and water for uptake as part of each minimal growth medium.

Supplementary Table S2: List of carbon sources that gave flux vectors with a nonzero growth rate under minimal media for aerobic conditions in the *S. cerevisiae* metabolic network (version iND750; Duarte *et*. *al*. *Genome Res*. **14**: 1298 (2004))

Abbreviation	Metabolite name	Abbreviation	Metabolite name
13BDglcn	1,3-beta-D-Glucan	glyc	Glycerol
4abut	4-Aminobutanoate	gsn	Guanosine
ac	Acetate	ins	Inosine
acald	Acetaldehyde	mal-L	L-Malate
adn	Adenosine	malt	Maltose
akg	2-Oxoglutarate	man	D-Mannose
ala-L	L-Alanine	melib	Melibiose
amet	S-Adenosyl-L-methionine	orn	Ornithine
arg-L	L-Arginine	pap	Adenosine 3',5'-bisphosphate
asn-L	L-Asparagine	pro-L	L-Proline
asp-L	L-Aspartate	pyr	Pyruvate
cit	Citrate	rib-D	D-Ribose
cytd	Cytidine	sbt-D	D-Sorbitol
etoh	Ethanol	ser-L	L-Serine
fru	D-Fructose	succ	Succinate
fum	Fumarate	sucr	Sucrose
gal	D-Galactose	tre	Trehalose
gam6p	D-Glucosamine 6-phosphate	uri	Uridine
glc-D	D-Glucose	xtsn	Xanthosine
gln-L	L-Glutamine	xyl-D	D-Xylose
glu-L	L-Glutamate	xylt	Xylitol
gly	Glycine		

Each carbon source was provided along with ammonium, oxygen, potassium, phosphate, proton, sodium, sulfate and water for uptake as part of each minimal growth medium.

Supplementary Table S3: List of carbon sources that gave flux vectors with a non zero growth under minimal media for aerobic conditions in the *S. aureus* metabolic network (version iSB619; Becker and Palsson, *BMC Microbiology* **5**: 8 (2005))

Abbreviation	Metabolite name	Abbreviation	Metabolite name
acgam	N-Acetyl-D-glucosamine	lac-D	D-Lactate
acnam	N-Acetylneuraminate	lac-L	L-Lactate
ala-D	D-Alanine	lcts	Lactose
ala-L	L-Alanine	malt	Maltose
arg-L	L-Arginine	man	D-Mannose
asp-L	L-Aspartate	mnl	D-Mannitol
etoh	Ethanol	orn	Ornithine
fru	D-Fructose	pro-L	L-Proline
glc-D	D-Glucose	rib-D	D-Ribose
glcn	D-Gluconate	ser-L	L-Serine
glu-L	L-Glutamate	sucr	Sucrose
glyc	Glycerol	thr-L	L-Threonine
glyc3p	Glycerol 3-phosphate	tre	Trehalose
hdca	Hexadecanoate (n-C16:0)		

Each carbon source was provided along with ammonium, Cu2+, cytosine, Fe2+, Mg, Mn2+, Ni2+, nicotinate, nitrate, nitrite, oxygen, phosphate, potassium, proton, sodium, sulfate, thiamin, water for uptake as part of each minimal growth medium.

Supplementary Table S4: UP(UC) reaction statistics in the original and reduced metabolic networks

**Organism**	***E. coli***	***S. cerevisiae***	***S. aureus***
Number of reactions in the original network	1176	1579	865
Number of UP reactions in the original network	289	391	277
Number of UC reactions in the original network	272	370	218
Number of UP/UC reactions in the original network	417	583	376
Number of blocked reactions	290	800	294
Number of UP/UC reactions in the original network that are blocked	136	386	174
Number of reactions in the reduced network	886	779	571
Number of UP reactions in the reduced network	245	218	224
Number of UC reactions in the reduced network	245	218	181
Number of UP/UC reactions in the reduced network	352	306	276
Number of UP/UC reactions in the reduced network that are not UP/UC in the original network	71	109	74

Supplementary Table S5: Size distribution of UP-UC clusters in *E. coli* network and its randomized versions. 85 UP-UC clusters of size ranging from 2 to 10 reactions were found in the network; the number of clusters of each size is given in the second column of the table. The third column gives the UP-UC cluster size distribution for randomized networks with same local connectivity as the real network, averaged over 1000 realizations of the randomized network.

Size of UP-UC cluster	Number of clusters in real network	Number of clusters in randomized networks Mean ± S.D.
2	49	101.32 ± 8.60
3	16	22.49 ± 4.28
4	5	6.62 ± 2.38
5	7	2.15 ± 1.44
6	2	0.84 ± 0.89
7	0	0.34 ± 0.57
8	2	0.15 ± 0.39
9	3	0.06 ± 0.24
10	1	0.02 ± 0.14

Supplementary Table S6: UP-UC clusters of metabolic reactions and the clustering of their genes in operons

The table lists the 85 UP-UC clusters of various sizes in *E. coli* metabolic network, computed as described in the main text. The network used is the one compiled by Reed *et al*. *Genome Biology* **4:** R54.1 (2003). In the table below, the abbreviations of reactions and metabolites, the description of the metabolic pathway where the reaction occurs, the reaction equation and Gene-Protein-Reaction (GPR) association is taken from the same database. After identifying the gene(s) for each reaction in every cluster using the GPR association, we determined which of the genes associated with a cluster are in the same operon. For a given cluster, genes in the same operon are coloured with the same shade (red, brown or pink). Information about operons was obtained from RegulonDB (Salgado *et al*. *Nucleic Acid Res.* **32**, D303 (2003)) and Ecocyc (Karp *et al*. *Nucleic Acid Res.* **30**, 56 (2002)).

Furthermore, we have added information obtained using flux balance analysis as to whether each reaction is 'active' or 'inactive'. Active reactions are those that were found to have a non-zero flux for at least one of the 89 flux vectors each corresponding to a different minimal medium (see main text), and inactive reactions are those that had a zero flux for all the 89 flux vectors. Reactions for which the corresponding gene name was not available in the database have been labelled as NA in the GPR association column. Of these 85 UP-UC clusters, 69 clusters are such that genes are identified for at least two distinct reactions in the cluster. The remaining 16 clusters include (a) 8 clusters that do not have at least two identified genes in the network, and (b) 8 forward and reverse direction pairs of the same reversible reaction. Such clusters are shaded blue. Of the above 69 clusters, 42 show a significant level of coregulation in that two or more genes in the cluster are part of the same operon. Of these 42, 26 are in the active set and 16 are in the inactive set.

SIZE TWO CLUSTERS

S. No.	Category	Abbreviation	GPR Association	Description	Reaction
1	Active	BUTCT	(b2221 and b2222)	Alternate Carbon Metabolism	accoa + but --> ac + btcoa
1	Active	FAO4	(b3846 and b0221)	Alternate Carbon Metabolism	btcoa + fad + h2o + nad --> aacoa + fadh2 + h + nadh
2	Active	GALCTND	b3692	Alternate Carbon Metabolism	galctn-D --> 2dh3dgal + h2o
2	Active	DDGALK	b3693	Alternate Carbon Metabolism	2dh3dgal + atp --> 2dh3dgal6p + adp + h
3	Inactive	FRUK	b2168	Alternate Carbon Metabolism	atp + f1p --> adp + fdp + h
3	Inactive	FRUpts	(b2167 and b2169 and b2415 and b2416)	Transport, Extracellular	fru[e] + pep --> f1p + pyr
4	Active	DHPPD	b2541	Alternate Carbon Metabolism	cechddd + nad --> dhpppn + h + nadh
4	Active	PPPNDO	(b2538 and b2539 and b2540 and b2542)	Alternate Carbon Metabolism	h + nadh + o2 + pppn --> cechddd + nad
5	Inactive	DHCIND	b2541	Alternate Carbon Metabolism	cenchddd + nad --> dhcinnm + h + nadh
5	Inactive	CINNDO	(b2538 and b2539 and b2540 and b2542)	Alternate Carbon Metabolism	cinnm + h + nadh + o2 --> cenchddd + nad
6	Active	GALS3	b4119	Alternate Carbon Metabolism	h2o + melib --> gal + glc-D
6	Active	MELIBt2	b4120	Transport, Extracellular	h[e] + melib[e] --> h + melib
7	Active	DHCINDO	b0348	Alternate Carbon Metabolism	dhcinnm + o2 --> hkntd
7	Active	HKNTDH	b0349	Alternate Carbon Metabolism	h2o + hkntd --> fum + (2) h + op4en
8	Active	HPPPNDO	b0348	Alternate Carbon Metabolism	dhpppn + o2 --> hkndd
8	Active	HKNDDH	b0349	Alternate Carbon Metabolism	h2o + hkndd --> (2) h + op4en + succ
9	Active	OP4ENH	b0350	Alternate Carbon Metabolism	h2o + op4en --> 4h2opntn
9	Active	HOPNTAL	b0352	Alternate Carbon Metabolism	4h2opntn --> acald + pyr
10	Inactive	TRE6PP	b1897	Alternate Carbon Metabolism	h2o + tre6p --> pi + tre
10	Inactive	TREH	b3519	Alternate Carbon Metabolism	h2o + tre --> (2) glc-D
11	Active	RBK	b3752	Alternate Carbon Metabolism	atp + rib-D --> adp + h + r5p
11	Active	RIBabc	((b4231 and b4227 and b4228 and b4229 and b4230) or (b3749 and b3751 and b3750 and b3748))	Transport, Extracellular	atp + h2o + rib-D[e] --> adp + h + pi + rib-D
12	Inactive	KG6PDC	(b3581 or b4196)	Alternate Carbon Metabolism	3dgulnp + h --> co2 + xu5p-L
12	Inactive	X5PL3E	b4197	Alternate Carbon Metabolism	xu5p-L --> ru5p-L
13	Inactive	TAUDO	b0368	Alternate Carbon Metabolism	akg + o2 + taur --> aacald + co2 + h + so3 + succ
13	Inactive	TAURabc	(b0365 and b0366 and b0367)	Transport, Extracellular	atp + h2o + taur[e] --> adp + h + pi + taur

14	Active	G5SD	b0243	Arginine and Proline Metabolism	glu5p + h + nadph --> glu5sa + nadp + pi
14	Active	GLU5K	b0242	Arginine and Proline Metabolism	atp + glu-L --> adp + glu5p
15	Active	ARGDC	(b2938 or b4117)	Arginine and Proline Metabolism	arg-L + h --> agm + co2
15	Active	AGMT	b2937	Arginine and Proline Metabolism	agm + h2o --> ptrc + urea
16	Inactive	ETHAAL	(b2440 and b2441)	Cell Envelope Biosynthesis	etha --> acald + nh4
16	Inactive	GPDDA2	b2239	Cell Envelope Biosynthesis	g3pe + h2o --> etha + glyc3p + h
17	Active	UHGADA	b0096	Cell Envelope Biosynthesis	h2o + u3aga --> ac + u3hga
17	Active	U23GAAT	b0179	Cell Envelope Biosynthesis	3hmrsACP + u3hga --> ACP + h + u23ga
18	Inactive	EDTXS3	b2378	Cell Envelope Biosynthesis	hdeACP + kdo2lipid4 --> ACP + kdo2lipid4p
18	Inactive	EDTXS4	b1855	Cell Envelope Biosynthesis	kdo2lipid4p + myrsACP --> ACP + lipa_cold
19	Inactive	G1PTT	(b3789 or b2039)	Cell Envelope Biosynthesis	dttp + g1p + h --> dtdpglu + ppi
19	Inactive	TDPGDH	(b3788 or b2041)	Cell Envelope Biosynthesis	dtdpglu --> dtdp4d6dg + h2o
20	Inactive	TDPDRE	b2038	Cell Envelope Biosynthesis	dtdp4d6dg --> dtdp4d6dm
20	Inactive	TDPDRR	b2040	Cell Envelope Biosynthesis	dtdp4d6dm + h + nadph --> dtdprmn + nadp
21	Inactive	GLUCYS	b2638	Cofactor and Prosthetic Group Biosynthesis	atp + cys-L + glu-L --> adp + glucys + h + pi
21	Inactive	GTHS	b2947	Cofactor and Prosthetic Group Biosynthesis	atp + glucys + gly --> adp + gthrd + h + pi
22	Active	QULNS	b0750	Cofactor and Prosthetic Group Biosynthesis	dhap + iasp --> h + (2) h2o + pi + quln
22	Active	NNDPR	b0109	Cofactor and Prosthetic Group Biosynthesis	(2) h + prpp + quln --> co2 + nicrnt + ppi
23	Active	NNAT	b0639	Cofactor and Prosthetic Group Biosynthesis	atp + h + nicrnt --> dnad + ppi
23	Active	NADS1	b1740	Cofactor and Prosthetic Group Biosynthesis	atp + dnad + nh4 --> amp + h + nad + ppi
24	Inactive	PMPK	b2103	Cofactor and Prosthetic Group Biosynthesis	4ampm + atp --> 2mahmp + adp
24	Inactive	TMPPP	b3993	Cofactor and Prosthetic Group Biosynthesis	2mahmp + 4mpetz + h --> ppi + thmmp
25	Active	GLYATi	b3617	Glycine and Serine Metabolism	2aobut + coa --> accoa + gly
25	Active	THRD	b3616	Glycine and Serine Metabolism	nad + thr-L --> 2aobut + h + nadh
26	Active	GLCS1	b3429	Glycolysis/Gluconeogenesis	adpglc --> adp + glycogen + h
26	Active	GLGC	b3430	Glycolysis/Gluconeogenesis	atp + g1p + h --> adpglc + ppi
27	Active	FAO3	(b3845 and b3846 and b1805 and b0221)	Membrane Lipid Metabolism	atp + (9) coa + (8) fad + (8) h2o + (8) nad + ocdca --> (9) accoa + amp + (8) fadh2 + (8) h + (8) nadh + ppi
27	Active	OCDCAt2	b2344	Transport, Extracellular	h[e] + ocdca[e] --> h + ocdca
28	Inactive	CYNTAH	b0340	Nitrogen	cynt + (3) h + hco3 --> (2) co2 + nh4
28	Inactive	CYNTt2	b0341	Putative Transporters	cynt[e] + h[e] --> cynt + h
29	Active	PRFGS	b2557	Purine and Pyrimidine Biosynthesis	atp + fgam + gln-L + h2o --> adp + fpram + glu-L + h + pi
29	Active	PRAIS	b2499	Purine and Pyrimidine Biosynthesis	atp + fpram --> adp + air + (2) h + pi
30	Inactive	LYSDC	(b4131 or b0186)	Threonine and Lysine Metabolism	h + lys-L --> 15dap + co2
30	Inactive	CADVt	b4132	Transport, Extracellular	15dap + h[e] + lys-L[e] --> 15dap[e] + h + lys-L
31	Active	HSK	b0003	Threonine and Lysine Metabolism	atp + hom-L --> adp + h + phom
31	Active	THRS	b0004	Threonine and Lysine Metabolism	h2o + phom --> pi + thr-L
32	Inactive	TSULabc	((b2425 and b2424 and b2423 and b2422) or (b2424 and b2423 and b2422 and b3917))	Transport, Extracellular	atp + h2o + tsul[e] --> adp + h + pi + tsul
32	Inactive	CYANST	b3425	Unassigned	cyan + tsul --> h + so3 + tcynt
33	Active	DHQS	b3389	Tyrosine, Tryptophan, and Phenylalanine Metabolism	2dda7p --> 3dhq + pi
33	Active	DDPA	(b2601 or b1704 or b0754)	Tyrosine, Tryptophan, and Phenylalanine Metabolism	e4p + h2o + pep --> 2dda7p + pi
34	Active	ACGS	b2818	Arginine and Proline Metabolism	accoa + glu-L --> acglu + coa + h
34	Active	ACGK	b3959	Arginine and Proline Metabolism	acglu + atp --> acg5p + adp
35	Active	UAGDP	b3730	Cell Envelope Biosynthesis	acgam1p + h + utp --> ppi + uacgam
35	Active	G1PACT	b3730	Cell Envelope Biosynthesis	accoa + gam1p --> acgam1p + coa + h
36	Inactive	GSPMDA	b2988	Arginine and Proline Metabolism	gtspmd + h2o --> gthrd + spmd
36	Inactive	GSPMDS	b2988	Arginine and Proline Metabolism	atp + gthrd + spmd --> adp + gtspmd + h + pi
37	Inactive	HYPOE	NA	Cofactor and Prosthetic Group Biosynthesis	h2o + pyam5p --> pi + pydam

37	Inactive	PYDAMK	b2418	Cofactor and Prosthetic Group Biosynthesis	atp + pydam --> adp + h + pyam5p
38	Inactive	PYDXPP	NA	Cofactor and Prosthetic Group Biosynthesis	h2o + pydx5p --> pi + pydx
38	Inactive	PYDXK	(b1636 or b2418)	Cofactor and Prosthetic Group Biosynthesis	atp + pydx --> adp + h + pydx5p
39	Inactive	PDXPP	NA	Cofactor and Prosthetic Group Biosynthesis	h2o + pdx5p --> pi + pydxn
39	Inactive	PYDXNK	b2418	Cofactor and Prosthetic Group Biosynthesis	atp + pydxn --> adp + h + pdx5p
40	Inactive	DMATT	b0421	Cofactor and Prosthetic Group Biosynthesis	dmpp + ipdp --> grdp + ppi
40	Inactive	GRTT	b0421	Cofactor and Prosthetic Group Biosynthesis	grdp + ipdp --> frdp + ppi
41	Active	PTRCTA	NA	Arginine and Proline Metabolism	akg + ptrc --> 4abutn + glu-L
41	Active	ABUTD	NA	Arginine and Proline Metabolism	4abutn + h2o + nad --> 4abut + (2) h + nadh
42	Inactive	AMAOTr	b0774	Cofactor and Prosthetic Group Biosynthesis	8aonn + amet --> amob + dann
42	Inactive	AMAOTr	b0774	Cofactor and Prosthetic Group Biosynthesis	amob + dann --> 8aonn + amet
43	Inactive	AOXSr	b0776	Cofactor and Prosthetic Group Biosynthesis	ala-L + h + pmcoa --> 8aonn + co2 + coa
43	Inactive	AOXSr	b0776	Cofactor and Prosthetic Group Biosynthesis	8aonn + co2 + coa --> ala-L + h + pmcoa
44	Inactive	CBIAT	b1270	Cofactor and Prosthetic Group Biosynthesis	atp + cbi + h2o --> adocbi + pi + ppi
44	Inactive	CBIAT	b1270	Cofactor and Prosthetic Group Biosynthesis	adocbi + pi + ppi --> atp + cbi + h2o
45	Inactive	GTHOr	b3500	Cofactor and Prosthetic Group Biosynthesis	gthox + h + nadph --> (2) gthrd + nadp
45	Inactive	GTHOr	b3500	Cofactor and Prosthetic Group Biosynthesis	(2) gthrd + nadp --> gthox + h + nadph
46	Inactive	ADK4	b0474	Nucleotide Salvage Pathways	amp + itp --> adp + idp
46	Inactive	ADK4	b0474	Nucleotide Salvage Pathways	adp + idp --> amp + itp
47	Inactive	TMDPP	b4382	Nucleotide Salvage Pathways	pi + thymd --> 2dr1p + thym
47	Inactive	TMDPP	b4382	Nucleotide Salvage Pathways	2dr1p + thym --> pi + thymd
48	Inactive	CRNBTCT	b0038	Oxidative phosphorylation	bbtcoa + crn --> crncoa + gbbtn
48	Inactive	CRNBTCT	b0038	Oxidative phosphorylation	crncoa + gbbtn --> bbtcoa + crn
49	Inactive	CRNCBCT	b0038	Oxidative phosphorylation	crn + ctbtcoa --> crncoa + ctbt
49	Inactive	CRNCBCT	b0038	Oxidative phosphorylation	crncoa + ctbt --> crn + ctbtcoa

SIZE THREE CLUSTERS

S. No.	Category	Abbreviation	GPR Association	Description	Reaction
1	Active	FFSD	NA	Alternate Carbon Metabolism	h2o + suc6p --> fru + g6p
1	Active	XYLI2i	b3565	Alternate Carbon Metabolism	fru --> glc-D
1	Active	SUCpts	(b2417 and b2415 and b2416 and b2429)	Transport, Extracellular	pep + sucr[e] --> pyr + suc6p
2	Active	MICITD	(b1276 or b0118)	Alternate Carbon Metabolism	2mcacn + h2o --> micit
2	Active	MCITS	b0333	Alternate Carbon Metabolism	h2o + oaa + ppcoa --> 2mcit + coa + h
2	Active	MCITD	b0334	Alternate Carbon Metabolism	2mcit --> 2mcacn + h2o
3	Inactive	ALDD19x	b1385	Alternate Carbon Metabolism	h2o + nad + pacald --> (2) h + nadh + pac
3	Inactive	PACCOAL	b1398	Alternate Carbon Metabolism	atp + coa + pac --> amp + phaccoa + ppi
3	Inactive	PEAMNO	b1386	Alternate Carbon Metabolism	h2o + o2 + peamn --> h2o2 + nh4 + pacald
4	Active	ACNML	b3225	Alternate Carbon Metabolism	acnam --> acmana + pyr
4	Active	AMANK	b3222	Putative	acmana + atp --> acmanap + adp + h
4	Active	ACNAMt2	b3224	Transport, Extracellular	acnam[e] + h[e] --> acnam + h
5	Active	MTRK	NA	Arginine and Proline Metabolism	5mtr + atp --> 5mdr1p + adp + h
5	Active	MTAN	b0159	Arginine and Proline Metabolism	5mta + h2o --> 5mtr + ade
5	Active	SPMS	b0121	Arginine and Proline Metabolism	ametam + ptrc --> 5mta + h + spmd
6	Active	KDOPP	NA	Cell Envelope Biosynthesis	h2o + kdo8p --> kdo + pi
6	Active	KDOPS	b1215	Cell Envelope Biosynthesis	ara5p + h2o + pep --> kdo8p + pi
6	Active	KDOCT2	b0918	Cell Envelope Biosynthesis	ctp + kdo --> ckdo + ppi
7	Inactive	DHPTDC	NA	Methionine Metabolism	dhptd --> h2o + hmfurn
7	Inactive	RHCCE	b2687	Methionine Metabolism	rhcys --> dhptd + hcys-L

7	Inactive	AHCYSNS	b0159	Methionine Metabolism	ahcys + h2o --> ade + rhcys
8	Active	HSST	b4013	Methionine Metabolism	hom-L + succoa --> coa + suchms
8	Active	SHSL1	b3939	Methionine Metabolism	cys-L + suchms --> cyst-L + h + succ
8	Active	CYSTL	(b3008 or b1662)	Methionine Metabolism	cyst-L + h2o --> hcys-L + nh4 + pyr
9	Inactive	LGTHL	b1651	Methylglyoxal Metabolism	gthrd + mthgxl --> lgt-S
9	Inactive	GLYOX	b0212	Methylglyoxal Metabolism	h2o + lgt-S --> gthrd + h + lac-D
9	Inactive	MGSA	b0963	Methylglyoxal Metabolism	dhap --> mthgxl + pi
10	Active	UGLYCH	b0505	Nitrogen	(2) h + h2o + urdglyc --> co2 + glx + (2) nh4
10	Active	ALLTN	b0512	Nitrogen	alltn + h2o --> alltt + h
10	Active	ALLTAH	b0516	Nitrogen	alltt + h2o --> urdglyc + urea
11	Active	DHDPS	b2478	Threonine and Lysine Metabolism	aspsa + pyr --> 23dhdp + h + (2) h2o
11	Active	DHDPRy	b0031	Threonine and Lysine Metabolism	23dhdp + h + nadph --> nadp + thdp
11	Active	THDPS	b0166	Threonine and Lysine Metabolism	h2o + succoa + thdp --> coa + sl2a6o
12	Active	ACHBS	((b3670 and b3671) or (b3769 and b3768 and b3767) or (b0077 and b0078))	Valine, leucine, and isoleucine metabolism	2obut + h + pyr --> 2ahbut + co2
12	Active	KARA2i	b3774	Valine, leucine, and isoleucine metabolism	2ahbut + h + nadph --> 23dhmp + nadp
12	Active	DHAD2	b3771	Valine, leucine, and isoleucine metabolism	23dhmp --> 3mop + h2o
13	Active	ACLS	((b3670 and b3671) or (b3769 and b3768 and b3767) or (b0077 and b0078))	Valine, leucine, and isoleucine metabolism	h + (2) pyr --> alac-S + co2
13	Active	KARA1i	b3774	Valine, leucine, and isoleucine metabolism	alac-S + h + nadph --> 23dhmb + nadp
13	Active	DHAD1	b3771	Valine, leucine, and isoleucine metabolism	23dhmb --> 3mob + h2o
14	Active	LEUTAi	(b4054 or b3770)	Valine, leucine, and isoleucine metabolism	4mop + glu-L --> akg + leu-L
14	Active	IPMD	b0073	Valine, leucine, and isoleucine metabolism	3c2hmp + nad --> 3c4mop + h + nadh
14	Active	OMCDC	b0073	Valine, leucine, and isoleucine metabolism	3c4mop + h --> 4mop + co2
15	Active	PGCD	b2913	Glycine and Serine Metabolism	3pg + nad --> 3php + h + nadh
15	Active	PSP_L	b4388	Glycine and Serine Metabolism	h2o + pser-L --> pi + ser-L
15	Active	PSERT	b0907	Glycine and Serine Metabolism	3php + glu-L --> akg + pser-L
16	Inactive	SHCHD2	b3368	Cofactor and Prosthetic Group Biosynthesis	nad + shcl --> h + nadh + srch
16	Inactive	SHCHF	b3368	Cofactor and Prosthetic Group Biosynthesis	fe2 + srch --> (3) h + sheme
16	Inactive	UPP3MT	(b3368 or b3803)	Cofactor and Prosthetic Group Biosynthesis	(2) amet + uppg3 --> (2) ahcys + h + shcl

SIZE FOUR CLUSTERS

S. No.	Category	Abbreviation	GPR Association	Description	Reaction
1	Inactive	GDMANE	b2052	Cell Envelope Biosynthesis	gdpddman --> gdpofuc
1	Inactive	GOFUCR	b2052	Cell Envelope Biosynthesis	gdpofuc + h + nadph --> gdpfuc + nadp
1	Inactive	GMAND	b2053	Cell Envelope Biosynthesis	gdpmann --> gdpddman + h2o
1	Inactive	MAN1PT2	b2049	Cell Envelope Biosynthesis	gdp + h + man1p --> gdpmann + pi
2	Active	PMDPHT	NA	Cofactor and Prosthetic Group Biosynthesis	5aprbu + h2o --> 4r5au + pi
2	Active	GTPCII2	b1277	Cofactor and Prosthetic Group Biosynthesis	gtp + (3) h2o --> 25drapp + for + (2) h + ppi
2	Active	APRAUR	b0414	Cofactor and Prosthetic Group Biosynthesis	5apru + h + nadph --> 5aprbu + nadp
2	Active	DHPPDA2	b0414	Cofactor and Prosthetic Group Biosynthesis	25drapp + h + h2o --> 5apru + nh4
3	Active	MOHMT	b0134	Cofactor and Prosthetic Group Biosynthesis	3mob + h2o + mlthf --> 2dhp + thf
3	Active	PANTS	b0133	Cofactor and Prosthetic Group Biosynthesis	ala-B + atp + pant-R --> amp + h + pnto-R + ppi
3	Active	ASP1DC	b0131	Cofactor and Prosthetic Group Biosynthesis	asp-L + h --> ala-B + co2
3	Active	DPR	(b0425 or b3774)	Cofactor and Prosthetic Group Biosynthesis	2dhp + h + nadph --> nadp + pant-R
4	Active	ADSK	b2750	Cysteine Metabolism	aps + atp --> adp + h + paps
4	Active	SADT2	(b2751 and b2752)	Cysteine Metabolism	atp + gtp + h2o + so4 --> aps + gdp + pi + ppi
4	Active	PAPSR	b2762	Cysteine Metabolism	paps + trdrd --> (2) h + pap + so3 + trdox

4	Active	SULabc	((b2425 and b2422 and b2423 and b2424) or (b2424 and b2422 and b2423 and b2413 and b3917))	Transport, Extracellular	atp + h2o + so4[e] --> adp + h + pi + so4
5	Active	PRAIi	b1262	Tyrosine, Tryptophan, and Phenylalanine Metabolism	pran --> 2cpr5p
5	Active	IGPS	b1262	Tyrosine, Tryptophan, and Phenylalanine Metabolism	2cpr5p + h --> 3ig3p + co2 + h2o
5	Active	ANS	(b1263 and b1264)	Tyrosine, Tryptophan, and Phenylalanine Metabolism	chor + gln-L --> anth + glu-L + h + pyr
5	Active	ANPRT	b1263	Tyrosine, Tryptophan, and Phenylalanine Metabolism	anth + prpp --> ppi + pran

SIZE FIVE CLUSTERS

S. No.	Category	Abbreviation	GPR Association	Description	Reaction
1	Active	AST	b1747	Arginine and Proline Metabolism	arg-L + succoa --> coa + h + sucarg
1	Active	SOTA	b1745	Arginine and Proline Metabolism	akg + sucorn --> glu-L + sucgsa
1	Active	SADH	b1748	Arginine and Proline Metabolism	(2) h + (2) h2o + sucarg --> co2 + (2) nh4 + sucorn
1	Active	SGSAD	b1746	Arginine and Proline Metabolism	h2o + nad + sucgsa --> (2) h + nadh + sucglu
1	Active	SGDS	b1744	Arginine and Proline Metabolism	h2o + sucglu --> glu-L + succ
2	Active	MOAT	b3633	Cell Envelope Biosynthesis	ckdo + lipidA --> cmp + h + kdolipid4
2	Active	MOAT2	b3633	Cell Envelope Biosynthesis	ckdo + kdolipid4 --> cmp + h + kdo2lipid4
2	Active	LPADSS	b0182	Cell Envelope Biosynthesis	lipidX + u23ga --> h + lipidAds + udp
2	Active	TDSK	b0915	Cell Envelope Biosynthesis	atp + lipidAds --> adp + h + lipidA
2	Active	USHD	b0480	Cell Envelope Biosynthesis	h2o + u23ga --> (2) h + lipidX + ump
3	Active	PPCDC	NA	Cofactor and Prosthetic Group Biosynthesis	4ppcys + h --> co2 + pan4p
3	Active	DPCOAK	NA	Cofactor and Prosthetic Group Biosynthesis	atp + dpcoa --> adp + coa + h
3	Active	PPNCL2	NA	Cofactor and Prosthetic Group Biosynthesis	4ppan + ctp + cys-L --> 4ppcys + cmp + h + ppi
3	Active	PNTK	b3974	Cofactor and Prosthetic Group Biosynthesis	atp + pnto-R --> 4ppan + adp + h
3	Active	PTPATi	b3634	Cofactor and Prosthetic Group Biosynthesis	atp + h + pan4p --> dpcoa + ppi
4	Inactive	ADOCBLS	b1992	Cofactor and Prosthetic Group Biosynthesis	agdpcbi + rdmbzi --> adocbl + gmp + h
4	Inactive	NNDMBRT	b1991	Cofactor and Prosthetic Group Biosynthesis	dmbzid + nicrnt --> 5prdmbz + h + nac
4	Inactive	ADOCBIK	b1993	Cofactor and Prosthetic Group Biosynthesis	adocbi + atp --> adocbip + adp + h
4	Inactive	ACBIPGT	b1993	Cofactor and Prosthetic Group Biosynthesis	adocbip + gtp + h --> agdpcbi + ppi
4	Inactive	RZ5PP	b0638	Cofactor and Prosthetic Group Biosynthesis	5prdmbz + h2o --> pi + rdmbzi
5	Inactive	HEMEOS	b0428	Cofactor and Prosthetic Group Biosynthesis	frdp + h2o + pheme --> hemeO + ppi
5	Inactive	UPPDC1	b3997	Cofactor and Prosthetic Group Biosynthesis	(4) h + uppg3 --> (4) co2 + cpppg3
5	Inactive	CPPPGO	b2436	Cofactor and Prosthetic Group Biosynthesis	cpppg3 + (2) h + o2 --> (2) co2 + (2) h2o + pppg9
5	Inactive	PPPGO	b3850	Cofactor and Prosthetic Group Biosynthesis	(1.5) o2 + pppg9 --> (3) h2o + ppp9
5	Inactive	FCLT	b0475	Cofactor and Prosthetic Group Biosynthesis	fe2 + ppp9 --> (2) h + pheme
6	Inactive	DXPRIi	b0173	Cofactor and Prosthetic Group Biosynthesis	dxyl5p + h + nadph --> 2me4p + nadp
6	Inactive	MECDPDH	b2515	Cofactor and Prosthetic Group Biosynthesis	2mecdp + h --> h2mb4p + h2o
6	Inactive	MEPCT	b2747	Cofactor and Prosthetic Group Biosynthesis	2me4p + ctp + h --> 4c2me + ppi
6	Inactive	CDPMEK	b1208	Cofactor and Prosthetic Group Biosynthesis	4c2me + atp --> 2p4c2me + adp + h
6	Inactive	MECDPS	b2746	Cofactor and Prosthetic Group Biosynthesis	2p4c2me --> 2mecdp + cmp
7	Active	DB4PS	b3041	Cofactor and Prosthetic Group Biosynthesis	ru5p-D --> db4p + for + h
7	Active	RBFSa	b1662	Cofactor and Prosthetic Group Biosynthesis	4r5au + db4p --> dmlz + (2) h2o + pi
7	Active	RBFK	b0025	Cofactor and Prosthetic Group Biosynthesis	atp + ribflv --> adp + fmn + h
7	Active	FMNAT	b0025	Cofactor and Prosthetic Group Biosynthesis	atp + fmn + h --> fad + ppi
7	Active	RBFSb	b0415	Cofactor and Prosthetic Group Biosynthesis	(2) dmlz --> 4r5au + ribflv

SIZE SIX CLUSTERS

S. No.	Category	Abbreviation	GPR Association	Description	Reaction
1	Inactive	GLUTRS	b2400	Cofactor and Prosthetic Group Biosynthesis	atp + glu-L + trnaglu --> amp + glutrna + ppi
1	Inactive	GLUTRR	b1210	Cofactor and Prosthetic Group Biosynthesis	glutrna + h + nadph --> glu1sa + nadp + trnaglu
1	Inactive	PPBNGS	b0369	Cofactor and Prosthetic Group Biosynthesis	(2) 5aop --> h + (2) h2o + ppbng
1	Inactive	HMBS	b3805	Cofactor and Prosthetic Group Biosynthesis	h2o + (4) ppbng --> hmbil + (4) nh4
1	Inactive	UPP3S	b3804	Cofactor and Prosthetic Group Biosynthesis	hmbil --> h2o + uppg3
1	Inactive	G1SATi	b0154	Cofactor and Prosthetic Group Biosynthesis	glu1sa --> 5aop
2	Inactive	DHNAOT	b3930	Cofactor and Prosthetic Group Biosynthesis	dhna + octdp --> 2dmmq8 + co2 + h + ppi
2	Inactive	NPHS	b2262	Cofactor and Prosthetic Group Biosynthesis	sbzcoa --> coa + dhna
2	Inactive	SUCBZS	b2261	Cofactor and Prosthetic Group Biosynthesis	2shchc --> h2o + sucbz
2	Inactive	OXGDC2	b2264	Cofactor and Prosthetic Group Biosynthesis	akg + h + thmpp --> co2 + ssaltpp
2	Inactive	SHCHCS2	b2264	Cofactor and Prosthetic Group Biosynthesis	ichor + ssaltpp --> 2shchc + pyr + thmpp
2	Inactive	SUCBZL	b2260	Cofactor and Prosthetic Group Biosynthesis	atp + coa + sucbz --> amp + ppi + sbzcoa

SIZE EIGHT CLUSTERS

S. No.	Category	Abbreviation	GPR Association	Description	Reaction
1	Inactive	ECAP_EC	NA	Cell Envelope Biosynthesis	unagamuf --> eca_EC + h + udcpdp
1	Inactive	ACGAMT	b3784	Cell Envelope Biosynthesis	uacgam + udcpp --> ump + unaga
1	Inactive	UAG2Ei	b3786	Cell Envelope Biosynthesis	uacgam --> uacmam
1	Inactive	UACMAMO	b3787	Cell Envelope Biosynthesis	h2o + (2) nad + uacmam --> (3) h + (2) nadh + uacmamu
1	Inactive	TDPADGAT	b3790	Cell Envelope Biosynthesis	accoa + dtdp4addg --> coa + dtdp4aaddg + h
1	Inactive	TDPAGTA	b3791	Cell Envelope Biosynthesis	dtdp4d6dg + glu-L --> akg + dtdp4addg
1	Inactive	AADDGT	b3793	Cell Envelope Biosynthesis	dtdp4aaddg + unagamu --> dtdp + h + unagamuf
1	Inactive	ACMAMUT	b3794	Cell Envelope Biosynthesis	uacmamu + unaga --> h + udp + unagamu
2	Active	S7PI	b0222	Cell Envelope Biosynthesis	s7p --> gmhep7p
2	Active	GMHEPPA	b0200	Cell Envelope Biosynthesis	gmhep17bp + h2o --> gmhep1p + pi
2	Active	EDTXS1	b1054	Cell Envelope Biosynthesis	ddcaACP + kdo2lipid4 --> ACP + kdo2lipid4L
2	Active	EDTXS2	b1855	Cell Envelope Biosynthesis	kdo2lipid4L + myrsACP --> ACP + lipa
2	Active	AGMHE	b3619	Cell Envelope Biosynthesis	adphep-D,D --> adphep-L,D
2	Active	GMHEPAT	b3052	Cell Envelope Biosynthesis	atp + gmhep1p + h --> adphep-D,D + ppi
2	Active	GMHEPK	b3052	Cell Envelope Biosynthesis	atp + gmhep7p --> adp + gmhep17bp + h
2	Active	LPSSYN_EC	(b3620 and b3621 and b3622 and b3626 and b3627 and b3631)	Cell Envelope Biosynthesis	(3) adphep-L,D + (2) cdpea + (3) ckdo + lipa + (2) udpg --> (3) adp + (2) cdp + (3) cmp + (10) h + lps_EC + (2) udp

SIZE NINE CLUSTERS

S. No.	Category	Abbreviation	GPR Association	Description	Reaction
1	Active	PPTGS	NA	Cell Envelope Biosynthesis	uaagmda --> h + peptido_EC + udcpdp
1	Active	PAPPT3	b0087	Cell Envelope Biosynthesis	udcpp + ugmda --> uagmda + ump
1	Active	UAGCVT	b3189	Cell Envelope Biosynthesis	pep + uacgam --> pi + uaccg
1	Active	UAPGR	b3972	Cell Envelope Biosynthesis	h + nadph + uaccg --> nadp + uamr
1	Active	UAMAS	b0091	Cell Envelope Biosynthesis	ala-L + atp + uamr --> adp + h + pi + uama
1	Active	UAMAGS	b0088	Cell Envelope Biosynthesis	atp + glu-D + uama --> adp + h + pi + uamag
1	Active	UAAGDS	b0085	Cell Envelope Biosynthesis	26dap-M + atp + uamag --> adp + h + pi + ugmd

1	Active	UGMDDS	b0086	Cell Envelope Biosynthesis	alaala + atp + ugmd --> adp + h + pi + ugmda
1	Active	UAGPT3	b0090	Cell Envelope Biosynthesis	uacgam + uagmda --> h + uaagmda + udp
2	Inactive	HBZOPT	b4040	Cofactor and Prosthetic Group Biosynthesis	4hbz + octdp --> 3ophb + ppi
2	Inactive	OPHHX	b3835	Cofactor and Prosthetic Group Biosynthesis	2oph + (0.5) o2 --> 2ohph
2	Inactive	CHRPL	b4039	Cofactor and Prosthetic Group Biosynthesis	chor --> 4hbz + pyr
2	Inactive	OPHBDC	(b3843 or b2311)	Cofactor and Prosthetic Group Biosynthesis	3ophb + h --> 2oph + co2
2	Inactive	OMBZLM	b3833	Cofactor and Prosthetic Group Biosynthesis	2ombzl + amet --> 2ommbl + ahcys + h
2	Inactive	OMMBLHX	b0662	Cofactor and Prosthetic Group Biosynthesis	2ommbl + (0.5) o2 --> 2omhmbl
2	Inactive	OHPHM	b2232	Cofactor and Prosthetic Group Biosynthesis	2ohph + amet --> 2omph + ahcys + h
2	Inactive	DMQMT	b2232	Cofactor and Prosthetic Group Biosynthesis	2omhmbl + amet --> ahcys + h + q8h2
2	Inactive	OMPHHX	b2907	Cofactor and Prosthetic Group Biosynthesis	2omph + (0.5) o2 --> 2ombzl
3	Active	PRMICIi	b2024	Histidine Metabolism	prfp --> prlp
3	Active	IGPDH	b2022	Histidine Metabolism	eig3p --> h2o + imacp
3	Active	HISTP	b2022	Histidine Metabolism	h2o + hisp --> histd + pi
3	Active	HSTPT	b2021	Histidine Metabolism	glu-L + imacp --> akg + hisp
3	Active	HISTD	b2020	Histidine Metabolism	h2o + histd + (2) nad --> (3) h + his-L + (2) nadh
3	Active	IG3PS	(b2023 and b2025)	Histidine Metabolism	gln-L + prlp --> aicar + eig3p + glu-L + h
3	Active	ATPPRT	b2019	Histidine Metabolism	atp + prpp --> ppi + prbatp
3	Active	PRATPP	b2026	Histidine Metabolism	h2o + prbatp --> h + ppi + prbamp
3	Active	PRAMPC	b2026	Histidine Metabolism	h2o + prbamp --> prfp

SIZE TEN CLUSTERS

S. No.	Category	Abbreviation	GPR Association	Description	Reaction
1	Active	DNMPPA	NA	Cofactor and Prosthetic Group Biosynthesis	dhpmp + h2o --> dhnpt + pi
1	Active	DHNPA2	b3058	Cofactor and Prosthetic Group Biosynthesis	dhnpt --> 6hmhpt + gcald
1	Active	DHFS	b2315	Cofactor and Prosthetic Group Biosynthesis	atp + dhpt + glu-L --> adp + dhf + pi
1	Active	GTPCI	b2153	Cofactor and Prosthetic Group Biosynthesis	gtp + h2o --> ahdt + for
1	Active	HPPK2	b0142	Cofactor and Prosthetic Group Biosynthesis	6hmhpt + atp --> 6hmhptpp + amp + h
1	Active	DHPS2	b3177	Cofactor and Prosthetic Group Biosynthesis	4abz + 6hmhptpp --> dhpt + h + ppi
1	Active	DNTPPA	(b1865 or b0099)	Cofactor and Prosthetic Group Biosynthesis	ahdt + h2o --> dhpmp + h + ppi
1	Active	ADCS	(b3360 and b1812)	Cofactor and Prosthetic Group Biosynthesis	chor + gln-L --> 4adcho + glu-L
1	Active	ADCL	b1096	Cofactor and Prosthetic Group Biosynthesis	4adcho --> 4abz + h + pyr
1	Active	GCALDD	b1415	Folate Metabolism	gcald + h2o + nad --> glyclt + (2) h + nadh

Supplementary Table S7: The size distribution of correlated reaction sets (or 'co-sets') in the *E. coli* metabolic network as reported by Reed and Palsson (*Genome Research* **14**:1797-1805 (2004)) and their explanation in terms of UP-UC clusters.

Size of co-sets	Number of co-sets	Number of co-sets explained by UP-UC clusters	Breakup of explained co-sets into UP-UC clusters in the reduced network
2	39	22	22x(2)
3	12	8	7x(3) + 1x(2)
4	10	10	7x(4) + 2x(3) + 1x(2+2)
5	3	2	1x(4) + 1x(3+2)
9	2	2	1x(6+2) + 1x(4+2)

The notation used in this table is the same as that in Table 2 of the main text. It is evident from the fourth column that the bulk of reactions in the co-sets are constituted by UP-UC clusters.

We comment here on the similarities and differences between the perfect clusters mentioned in the main text and correlated reaction sets described by Reed and Palsson. Both approaches identify groups of reactions in the network that respond in a ‘similar’ or correlated fashion across multiple flux vectors. The clusters obtained from them overlap significantly. 58 of the 66 co-sets found by Reed and Palsson either exactly match our perfect clusters or differ from them by one or two reactions.

There are two main differences between the two methods.

First, Reed and Palsson use ‘binarized’ flux vectors whose entries only carry information about whether a reaction has zero or nonzero flux in a given flux vector. The correlation coefficient we use (given in the Methods section of the main text) has, on the other hand, the actual flux values of the reactions. Thus while the co-sets of Reed and Palsson would cluster together a pair of reactions that are always off together and on together, such a pair would have perfect correlation in our case only if it satisfied the further requirement that the fluxes of the two reactions were proportional to each other across all flux vectors (with the same proportionality constant). In this sense our definition of perfect clusters is tighter than that of co-sets. Thus, for example, reactions O2t, NADH6, CYTBO3, SUCD1i and DKMPPD are part of the same co-set in the case of Reed and Palsson, while they are not perfectly clustered according to our definition.

Second, we have only one flux vector for each feasible minimal medium in aerobic conditions, and hence only 89 flux vectors for E. coli, over which to determine the correlation coefficient. On the other hand Reed and Palsson have 56756 flux vectors across 136 growth conditions (88 aerobic and 48 anaerobic conditions). Two reactions that are perfectly correlated across a certain set of flux vectors may not be so if the set is enlarged. If a new flux vector is such that one of the pair has zero flux and the other a nonzero flux in the flux vector, then the pair would not be part of the same co-set. Thus by considering a larger set of flux vectors, Reed and Palsson have obtained a smaller number of co-sets than the number of perfect clusters we have found. Furthermore, clusters found by Reed and Palsson are in most cases subsets of our perfect clusters.

Supplementary Table S8: The size distribution of perfect clusters in the *S. cerevisiae* metabolic network and their explanation in terms of UP-UC clusters.

Size of perfect clusters	Number of perfect clusters	Number of perfect clusters explained	Breakup of explained clusters into UP-UC clusters in the reduced network
2	38	17	17x(2)
3	10	5	2x(3) + 3x(2)
4	8	4	3x(3) + 1x(2)
5	1	1	1x(5)
6	1	0	
7	1	0	
8	1	1	1x(8)
9	2	2	1x(7+2) + 1x(4)
10	1	0	
12	1	1	1x(4+2)
117	1	1	(18+9+7+7+6+6+5+4+3+3+3+3+3+3+2+2+2+2+2)

Supplementary Table S9: The size distribution of perfect clusters in the *S. aureus* metabolic network and their explanation in terms of UP-UC clusters.

Size of perfect clusters	Number of perfect clusters	Number of perfect clusters explained	Breakup of explained clusters into UP-UC clusters in the reduced network
2	23	8	8x(2)
3	11	6	4x(3) + 2x(2)
4	7	4	1x(4) + 2x(3) + 1x(2)
5	3	3	1x(5) + 1x(3) + 1x(2)
30	1	1	1x(28)
193	1	1	(9+9+8+7+7+6+6+6+5+5+5+4+4+4+4+4+3+3+3+3+3+2+2+2+2+2+2+2+2+2+2+2+2+2+2+2+2)
