# Supplementary material for: Low degree metabolites explain essential reactions and enhance modularity in biological networks
Source: BMC Bioinformatics. 2006 Mar 8;7:118. doi: 10.1186/1471-2105-7-118 (PMC1434774; doi:10.1186/1471-2105-7-118)
Supplement: Additional File 2 — Algorithm for computing UP-UC clusters in the metabolic network. [file 1471-2105-7-118-S2.pdf]

Additional File 2

for

"Low degree . . ." by A. Samal et al.

Algorithm for detecting UP-UC clusters in metabolic network

1. Starting from the reconstructed metabolic network of a organism, one prepares a list of metabolic reactions that has each reversible reaction in the original network replaced by two one sided reactions.

2. From this list of metabolic reactions, construct a matrix $A = (A_{i\alpha})$ of dimensions $n \times N$, where n is the total number of metabolites and N is the total number of reactions in the network. We set $A_{i\alpha}$ equal to -1 if metabolite i is consumed in reaction α, $+1$ if metabolite i is produced in reaction α, and 0 if metabolite i does not participate in reaction α.

3. A UP-UC metabolite has in-degree and out-degree equal to unity in the bipartite metabolic graph. Thus, metabolite i is UP-UC if the ith row of the matrix A has exactly one entry that equals -1, one entry that equals $+1$ and has all other entries 0. Furthermore, for the purpose of identifying UP-UC clusters, only internal metabolites are considered; external metabolites are not regarded as UP-UC even if they have in and out degree unity. This amounts to excluding the rows corresponding to the external metabolites from A matrix. The number of internal metabolites in the metabolic networks of *E. coli*, *S. cerevisiae* and *S. aureus* is 618, 945 and 561 respectively.

4. Delete all links in the graph except those going into or out of UP-UC metabolites, i.e., obtain a matrix B from A by setting every entry of each row in A that corresponds to a non UP-UC metabolite equal to 0.

5. From the matrix B, construct a reaction-reaction graph in which each node corresponds to a reaction. The adjacency matrix $C = (C_{\alpha\beta})$ of this graph is defined by $C_{\alpha\beta} = 1$ if $B_{i\alpha} = 1$ and $B_{i\beta} = -1$, else $C_{\alpha\beta} = 0$. The matrix C represents a directed graph.

6. The weak components of size ≥ 2 of the graph C are the various UP-UC clusters. These are determined as follows: First convert C into an undirected graph $\tilde{C}$ by dropping all the directions of the arrows, i.e., $\tilde{C}_{\alpha\beta} = 1$ if $C_{\alpha\beta} = 1$ or $C_{\beta\alpha} = 1$, else $\tilde{C}_{\alpha\beta} = 0$. Two nodes α and β in C are said to be weakly connected if there exists a path between them in $\tilde{C}$. A weak component is a maximal set of nodes that are weakly connected to each other.

A program for calculating UP-UC clusters is freely available from the authors.

Note that a choice has to be made as to whether to include or exclude the biomass reaction from the list of reactions in A and B. Its inclusion/exclusion gives slightly different results for the set of UP-UC metabolites and their clusters. The biomass reaction should be included in the A matrix in steps 1-3 above (identification of UP-UC metabolites), for if it is not, then those biomass metabolites which are consumed by only one reaction other than the biomass reaction get identified as UC metabolites resulting in some spurious UP-UC clusters. However in the B matrix (steps 4-6) it is a matter of convention whether the biomass reaction is included or not; results in the two cases are different but each is valid in its own right. The results quoted in the paper correspond to the following choice: In steps 1-3 above the A matrix includes the biomass reaction and in steps 4-6 the B matrix excludes it. When the biomass reaction is included in the B matrix the size of the largest UP-UC cluster increases.
